# Fabricating Patient-Specific 3D Printed Drill Guides to Treat Femoral Head Avascular Necrosis

**DOI:** 10.21203/rs.3.rs-3650115/v1

**Published:** 2023-12-04

**Authors:** Cameron E. Bell, Alborz Feizi, Gregory R. Roytman, Alim F. Ramji, Steven M. Tommasini, Daniel H. Wiznia

**Affiliations:** Yale University; Yale University; Yale University; Yale University; Yale University; Yale University

**Keywords:** Femur Head Necrosis, Osteonecrosis, Surgical Decompression, 3D Printing, Stereolithography, Computer-Aided Design

## Abstract

**Background:**

Femoral head avascular necrosis (AVN), or death of femoral head tissue due to a lack of blood supply, is a leading cause of total hip replacement for non-geriatric patients. Core decompression (CD) is an effective treatment to re-establish blood flow for patients with AVN. Techniques aimed at improving its efficacy are an area of active research. We propose the use of 3D printed drill guides to accurately guide therapeutic devices for CD.

**Methods:**

Using femur sawbones, image processing software, and 3D modeling software, we created a custom-built device with pre-determined drill trajectories and tested the feasibility of the 3D printed drill guides for CD. A fellowship trained orthopedic surgeon used the drill guide to position an 8 ga, 230 mm long decompression device in the three synthetic femurs. CT scans were taken of the sawbones with the drill guide and decompression device. CT scans were processed in the 3D modeling software. Descriptive statistics measuring the angular and needle-tip deviation were compared to the original virtually planned model.

**Results:**

Compared to the original 3D model, the trials had a mean displacement of 1.440±1.03 mm and a mean angle deviation of 1.093±0.749°.

**Conclusions:**

The drill guides were demonstrated to accurately guide the decompression device along its predetermined drill trajectory. Accuracy was assessed by comparing values to literature-reported values and considered AVN lesion size. This study demonstrates the potential use of 3D printing technology to improve the efficacy of CD techniques.

## INTRODUCTION

Avascular necrosis (AVN) is a painful and often debilitating ischemic disease that affects the health of the hip joint.^1^ The disease is characterized by interrupted blood supply to the subchondral region of the femoral head, leading to osteonecrosis and progressive degeneration. In advanced stages, AVN can result in femoral head collapse, which can only be ameliorated through total hip arthroplasty (THA).^2^

AVN often results in THA for patients under 50 years old, with reports suggesting it accounts for up to 46.9% of the performed procedures.^3, 4^ Due to the impact that THA exerts on the lifestyles of younger patients, numerous hip-restorative treatments have been developed over the years. Most notably core decompression (CD), the surgical removal of necrotic tissue by drilling into the femoral head,^1^ has been demonstrated to successfully delay femoral head degeneration when performed at the earliest stages of the disease.^5^ Efforts to improve the efficacy of CD procedures has been an active area of research. The use of surgical robots and computer navigation tools for CD have previously been described.^3^ However, such approaches can be expensive, time consuming, and are not employed in all hospitals.

The most common conventional technique is the freehand CD technique that aims to access lesions using fluoroscopic guidance. However, this technique has its limitations as well. Inadequate visualization of lesions and radiographs constrained to a single plane at a time compound the already-complex hand-eye coordination required to properly guide therapeutic devices. Rates of malposition of guidewires under fluoroscopy reportedly range between 2% and 15%.^3^

3D printing offers a novel, inexpensive solution to help surgeons accurately and precisely navigate therapeutic devices to intended targets. With the advent of 3D modeling software, patient CT scans can now form the basis of complex 3D renderings that allow for the creation of customizable, patient-specific medical instruments. Through surgical planning, these instruments can be tailored to the unique characteristics of each case.^6^ Moreover, the technology provides surgeons the opportunity to simulate any number of approaches before selecting the path that they deem to be most effective. In fact, 3D-printed drill guides have demonstrated accurate positioning of guide wires for the placement of percutaneous screws for femoral neck stabilization.^7^

The aim of this study was to design and determine the accuracy of patient-specific 3D-printed drill guides for CD. The accuracy of the 3D printed drill guide is a proof of concept for the proposed procedure. Upon further refinement, this approach has the potential to improve the treatment of AVN and represents the capabilities of 3D printing for personalized medicine.

## METHODS

A foam cortical shell femur (Model SKU 1103, Sawbones, Vashon, WA, USA) was scanned using a LightSpeed VCT GE Medical Systems CT Scanner with a slice thickness of 0.625 mm and 80 kVp. The CT scan was segmented using Synopsys Simpleware ScanIP ^®^ software (Version 2022, Sunnyvale, CA, USA), creating a 3D femur model. To represent the necrotic lesion, a virtual lesion was positioned in the head of the 3D femur model, which was based on previously described lesions in the literature.^2, 11, 12^

### Drill Guide Fabrication

The vastus ridge was selected as the site for the attachment of the drill guide because of its limited number of muscle attachments and its proximity to the conventional CD drill location. The device was constructed in our 3D modeling software by initially positioning a cylindrical geometry with dimensions of 30 mm in diameter and 30 mm in height near the vastus ridge. The final design of the drill guide was assembled by Boolean adding and Boolean subtracting geometries. A second cylinder measuring 30 mm in diameter by 5 mm in height was positioned over the anterior intertrochanteric crest and added to the main body to improve the attachment to the femur. To create a custom-fit device, portions of the device that intersected with the femur were subtracted and extraneous portions along the lateral shaft of the femur that would interfere with the attachment of the vastus lateralis were removed. A cone was added to support the guidewire. Spikes were added in the distal portion of the guide to perforate through the vastus lateralis and contact the lateral femur, ensuring the guide rested firmly along the lateral shaft of the femur.

**Figure 1:**
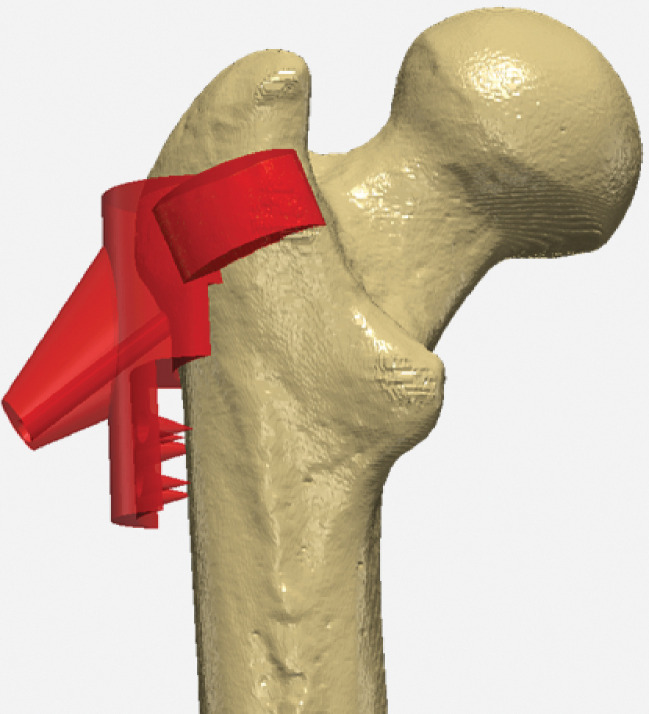
The first iteration of the prototype device imaged in ScanIP image processing software.

Subsequently, the drill trajectory was integrated into the device by subtracting a cylinder with dimensions of a conventional CD guidewire from the device. The cylindrical geometry with a diameter of 5 mm and length of 230 mm (based on the open-tip 8 ga × 230 mm delivery cannula, 7 mm IntraOsseous BioPlasty ^®^ decompression device, Arthrex, Naples, FL) was inserted into the model and positioned to traverse the femur and device with one face of the geometry centered in the lesion and the other exiting the precision cone of the guide. The final drill guide (Fig. 1) was 3D printed using the Form3B 3D Printer with Grey V4 resin (FormLabs, Somerville, MA, USA). Once printed, the drill guide was post-processed according to manufacturer guidelines.

To control for guide wire depth, a tube with an inner diameter of 2.6 mm, an outer diameter of 4.2 mm, and a length of 150 mm was printed to be placed over the distal end of the wire to prevent the guide wire from perforating the femoral head.

### Testing the Accuracy of the 3D-Printed Drill Guides

Three 3D printed drill guides were fixed to three foam cortical shell femurs. A fellowship-trained orthopedic surgeon drilled 4.1 mm diameter, 230 mm long Jamshidi needles into each of the foam femurs using a wire driver power system (Stryker, Kalamazoo, MI, USA). To confirm the accurate trajectory of the guidewire, post-drilling CT scans were obtained for the foam femurs.

### Comparison of Drilled Sawbones with Simulation Guide Wires

**Figure 2:**
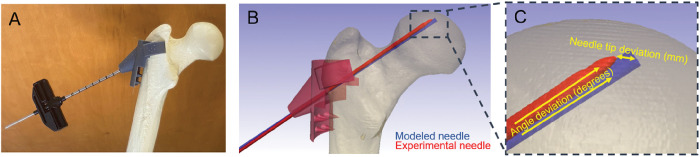
(A) 3D-printed guide fitted to a foam cortical shell femur is displayed. The femur was modeled using a dual-energy CT scan and the custom device was fitted using 3D modeling software. A Jamshidi needle, utilized subsequently for an AVN decompression device, was positioned using the modeled femur. A second dual-energy CT-scan was obtained after drilling the foam cortical shell femur with the Jamshidi needle. (B and C) The position of Jamshidi after drilling was compared to the 3D-modeled position.

The foam femur CT scans were rendered as 3D model masks using ScanIP image processing software. The 3D model masks from the accuracy tests were individually overlaid on the original modeled femur with the ideal drill trajectory (Fig. 2). The ScanIP measurement tool was used to find the positional deviation and angular deviation between theoretical and experimental drill trajectories. Positional deviation was determined by measuring the difference in drill tip location between the ideal drill trajectory and the test drill trajectories. Angular deviation was determined by measuring the angle between the guidewire and the ideal drill trajectory from the cortical entry point. Descriptive statistics (mean and range) were collected for the angle deviation and needle tip deviation.

## RESULTS

The mean angle deviation between the theoretical and measured needle tip was 1.093° (Range: 0.369° – 1.865°). The mean needle tip deviation between the theoretical and measured needle tip was 1.440 mm (Range: 0.257 mm – 2.149 mm). The standard deviation for the angle deviation and needle tip deviation were 0.749° and 1.03 mm, respectively. (Table 1)

## DISCUSSION

CD is an effective hip-preserving treatment for AVN at early stages of the disease. Moreover, variations to the CD techniques aimed at improving efficacy have been described and remain an area of active research. Our 3D printed drill guides demonstrated the ability to accurately guide therapeutic devices to the predetermined location. We assessed accuracy by comparing the results of our drill guides to those previously published in the literature and considered the size of AVN lesions. Our measured angle deviations were smaller than other 3D printed drill guides but was nonetheless consistent with the other deviations reportedly between 2.58° and 4.21°.^7, 8^

Furthermore, the needle tip deviations fell in the range of those previously reported and were compared to the relative to size of lesions. Steinberg et al. found thatpatients with necrotic lesions comprising less than 15% of the total volume of femoral head volume had significantly better outcomes when treated with CD.^9^ According to the average volume of the femoral head reported in Li et al.,our measured needle tip deviation would have been accurate when attempting to reach a volume < 7.395 cm^3^.^10^ Thus, despite deviations, our drill guides were determined to be accurate.^11, 12^

Using 3D printed drill guides for the CD procedure presents several potential advantages when compared to current techniques. Firstly, 3D printed guides offer a cost-effective alternative to robotic and computer navigated surgery while maintaining high level-accuracy. Our drill guides enhance the accuracy of therapeutic devices when compared to freehand drilling, thus increasing the likelihood of successful treatment. Moreover, the constrained drill trajectory reduces the need for radiographic images, thereby reducing patients’ exposure to radiation.

### Limitations

The creation of a femur-specific drill guide requires both accurate and precise femur models. The use of the foam cortical shell models simplified the modeling process for both the femur and drill-guide, however the process for modeling *in-vivo* femurs along with their respective drill guides may be more challenging. For instance, with the added complication of soft tissue small deviations in the attachment of the drill guide can translate to large deviations for the trajectory of the drilled needle.

An additional challenge to utilizing drill guides is the invasiveness of the procedure. To place the drill guide, the surgeon must make an incision and dissect down to the lateral greater trochanter to properly position the guide to the femur. The placement of the drill guide requires a large incision relative to conventional techniques, which increases patients’ risk of infection.

### Future Studies

Additional studies using cadaveric models are needed to validate the accuracy and effectiveness of the 3D printed drill guide. A direct comparison between the 3D printed drill guide and existing methods for CD is necessary to fully evaluate its potential benefits. Moreover, the feasibility of implementing the 3D printed drill guide in a clinical setting as well as potential risks and complications associated with its use should be considered.

## CONCLUSION

Our 3D printed drill guide prototype was determined to be accurate and reliable. Using this technique in surgical practice can provide several advantages over conventional techniques such as reducing time and thereby infection risk in CD surgery as well as lowering the cost of surgery. Although the 3D printed drill guide has the potential to improve the accuracy of core decompression procedures, further research is needed to fully evaluate its effectiveness and feasibility in a surgical setting.

## Figures and Tables

**Table 1:** The positional and angular deviation between each sample of the foam cortical femur compared to the ideal computer model of the foam cortical femur.

Sample	Angular Deviation (Degrees)	Positional Deviation (mm)
1	1.865	0.257
2	0.369	1.913
3	1.045	2.149

## Data Availability

Data used during this study are all included in the manuscript.

## Abbreviations

AVN: Avascular Necrosis
CD: Core Decompression
THA: Total Hip Arthroplasty
